# Supplementary material for: SARS-CoV-2 accessory proteins involvement in inflammatory and profibrotic processes through IL11 signaling
Source: Front Immunol. 2023 Jul 20;14:1220306. doi: 10.3389/fimmu.2023.1220306 (PMC10399023; doi:10.3389/fimmu.2023.1220306)
Supplement: Supplementary file 5 [file Table_5.docx]

**Table S5. List of antibodies**

| **Target protein** | **Supplier** | **Catalog number** |
| --- | --- | --- |
| Strep-Tag | Sigma | Cat#SAB2702215 |
| WNT5A | RD Systems | Cat#MAB645; RRID:AB_10571221 |
| c-jun | Cell Signaling | Cat#L70B11 |
| p-c-jun (S73) | Cell Signaling | Cat#D47G9 |
| STAT3 | abcam | Cat#ab280212 |
| pSTAT3 | abcam | Cat#ab76315; RRID:AB_1658549 |
| Smad2 | abcam | Cat#ab71109; RRID:AB_1281120 |
| pSmad2 | abcam | Cat#53100; RRID:AB_874025 |
| TGFb | abcam | Cat#ab215715; RRID:AB_2893156 |
| SERPINE1 (PAI-1) | abcam | Cat#ab222754 |
| GAPDH | Sigma | Cat#G8795; RRID:AB_1078991 |
| Phalloidin-FITC | Sigma | Cat#P5282 |
| Anti-mouse alexa 568 | Invitrogen | Cat#A-11004 |
| Anti-mouse-HRP | Sigma | Cat#A9044; RRID:AB_258431 |
| Anti-rabbit-HRP | abcam | Cat#ab6721; RRID:AB_955447 |
| Anti-rat-HRP | RD Systems | Cat#HAF005; RRID:AB_1512258 |
